# Carbon Availability Affects Diurnally Controlled Processes and Cell Morphology of *Cyanothece* 51142

**DOI:** 10.1371/journal.pone.0056887

**Published:** 2013-02-15

**Authors:** Jana Stöckel, Thanura R. Elvitigala, Michelle Liberton, Himadri B. Pakrasi

**Affiliations:** Department of Biology, Washington University, St. Louis, Missouri, United States of America; Missouri University of Science and Technology, United States of America

## Abstract

Cyanobacteria are oxygenic photoautotrophs notable for their ability to utilize atmospheric CO_2_ as the major source of carbon. The prospect of using cyanobacteria to convert solar energy and high concentrations of CO_2_ efficiently into biomass and renewable energy sources has sparked substantial interest in using flue gas from coal-burning power plants as a source of inorganic carbon. However, in order to guide further advances in this area, a better understanding of the metabolic changes that occur under conditions of high CO_2_ is needed. To determine the effect of high CO_2_ on cell physiology and growth, we analyzed the global transcriptional changes in the unicellular diazotrophic cyanobacterium *Cyanothece* 51142 grown in 8% CO_2_-enriched air. We found a concerted response of genes related to photosynthesis, carbon metabolism, respiration, nitrogen fixation, ribosome biosynthesis, and the synthesis of nucleotides and structural cell wall polysaccharides. The overall response to 8% CO_2_ in *Cyanothece* 51142 involves different strategies, to compensate for the high C/N ratio during both phases of the diurnal cycle. Our analyses show that high CO_2_ conditions trigger the production of carbon-rich compounds and stimulate processes such as respiration and nitrogen fixation. In addition, we observed that high levels of CO_2_ affect fundamental cellular processes such as cell growth and dramatically alter the intracellular morphology. This study provides novel insights on how diurnal and developmental rhythms are integrated to facilitate adaptation to high CO_2_ in *Cyanothece* 51142.

## Introduction

The significant increase in global energy consumption together with current and prospective economic growth is linked to severe concerns about rising CO_2_ emissions. CO_2_ is a greenhouse gas whose accumulation in the biosphere is related to potential global climate changes in the near future. CO_2_ is also the primary source of all organic carbon, and life on earth depends on the assimilation of CO_2_. Oxygenic photoautotrophic organisms such as cyanobacteria efficiently convert light into chemical energy using atmospheric CO_2_ and electrons from H_2_O while splitting off O_2_, and thus contribute significantly to the global carbon cycle. In addition, many cyanobacterial strains thrive when provided with higher levels of CO_2_ because the rate limiting enzyme ribulose bisphosphate carboxylase-oxygenase (Rubisco) has a low affinity for CO_2_ [1]. So far, the effects of CO_2_ on physiology and morphology in cyanobacteria have been primarily used to understand the mechanisms of carbon concentration under CO_2_ limiting conditions [2], [3], [4], [5].

Earlier studies in different diazotrophic cyanobacteria under elevated CO_2_ suggested a reallocation of energy otherwise utilized for carbon acquisition to other energy demanding processes such as nitrogen fixation and growth. Previous efforts to elucidate the impact of CO_2_ on cell physiology established the view that concentrations from 750–1500 ppm significantly increase nitrogen and carbon fixation rates [6], [7], [8], [9], [10]. These analyses were corroborated by measurements of elemental compositions and showed a correlation between cellular carbon quotas, nitrogen levels and population growth. In addition, transcriptional analysis in the non-diazotrophic cyanobacterium *Synechocystis* sp. 6803 revealed CO_2_-mediated changes in the expression of genes associated with photosynthesis, carbon fixation, and nitrate assimilation [2], [11]. Although these studies substantially aided our understanding about cyanobacterial physiology in elevated CO_2_ conditions, details about metabolic adaptations under high CO_2_ levels remain elusive.

Recently, in the advent of biofuel research, cyanobacteria received much attention for their potential to convert CO_2_ directly into renewable energy sources [12]. In addition, the idea of using cyanobacteria to capture and remediate anthropogenic CO_2_ substantiates the need to understand global cellular responses under high CO_2_ conditions.

The capacity to store large amounts of carbon in the form of glycogen, together with the ability to adjust nutritional requirements for nitrogen by converting atmospheric N_2_ into ammonia, renders *Cyanothece* 51142 an ideal candidate for studying the impact of high CO_2_ on cell physiology and metabolism. *Cyanothece* 51142 temporally separates and performs photosynthesis and N_2_ fixation in the same single cell. Oscillations in the activities of photosynthesis and N_2_ fixation occur concomitantly with the accumulation and degradation of glycogen and cyanophycin, and result in dynamic and diurnally controlled changes of the cellular C/N ratio. Previous global transcriptional analyses under alternating light/dark conditions revealed significant oscillations at the transcript level in about 30% of the genes in the genome [13]. Subsequent proteome analyzes showed that modifications in diverse cellular activities during the diurnal cycle can be attributed to changes in the abundance of related proteins [14]. In order to elucidate the impact of high CO_2_ on the metabolism of *Cyanothece* 51142 in alternating light/dark conditions, we analyzed the changes in gene expression under 8% CO_2_-enriched air, which is about 50% of the CO_2_ concentration found in flue gas exhausts [15]. We identified a total of 2086 differentially expressed genes. Approximately 40% (875 genes) revealed higher transcript levels under 8% CO_2_-enriched air. Our transcriptional analyses were complemented with different physiological and metabolic measurements to visualize novel aspects of the complex intracellular network that has developed to integrate carbon availability with cell metabolism and growth.

## Materials and Methods

### Cell Growth

The *Cyanothece* 51142 cultures were grown in 1 L Roux culture bottles (Bellco Glass, Inc.) containing ASP2 medium without added nitrate at pH 7.4 buffered with 4.1 mM TAPS and 3.9 mM TAPSO [16]. The cultures were kept under alternating 12 h light/dark cycles, illuminated with 100 µmol photons/m^2^/s of white light at 30 °C, and aerated with ambient CO_2_. Initially, the cultures were inoculated with 1/10 volume of cells grown under nitrogen-sufficient conditions and continuous illumination of 50 µmol photons/m^2^/s of white light. After 7 d, 1/4 volume of the nitrogen-deficient pre-culture was used to inoculate fresh ASP2 media without added nitrate and further cultivated under the same conditions. For microarray analysis, cultures were adapted to 8% CO_2_-enriched air for 48 h prior to the transition to ambient conditions or were kept in high CO_2_ during the entire time course. Samples from air and 8% CO_2_ treated cultures were collected 30 min, 2 h, 6 h, 13 h, 18 h, 30 h and 42 h after the step down corresponding to the time points, L0.5, L2, L6, D1, D6, L6, and D6.

### Glycogen assay

The cellular glycogen content was measured using a glucose hexokinase assay (Sigma-Aldrich, St. Louis, MO, USA) with glycogen from bovine liver Type IX (Sigma-Aldrich, St. Louis, MO, USA) as standard. Samples were collected at the time points L6 and D6. The chlorophyll was extracted with 100% methanol before the cell pellets were washed twice with 100% ethanol. 40% KOH was added and the samples were incubated for 1 h at 95 °C to remove free glucose. Glycogen was precipitated after adding 2 volumes of 100% ethanol, and the samples were incubated overnight at −20 °C. After centrifugation for 1 h at 4 °C, 2 N HCl was added, and the samples were incubated at 95 °C for 30 min. One volume 2N NaOH and 0.5 volumes 1 M phosphate buffer, pH 7, were added, and the samples were diluted with 1 volume of distilled water. For the hexokinase assay, 75 µl of sample solution was mixed with 200 µl of enzyme solution, and after 15 min incubation at ambient temperature in a microtitre plate (Costar, ultraviolet light proof); NADPH was measured at 340 nm using a μQuant plate reader (Bio-Tek Instruments).

### Free amino acid measurements

The samples were extracted from two biological replicates and measured as previously described [17].

### Nitrogenase assay

Nitrogenase activity of *Cyanothece* 51142 cultures grown under aeration with ambient and 8% CO_2_-enriched air was determined using an acetylene reduction assay [18]. 20 mL cultures were harvested at the end of the light period, transferred into gas tight vials and incubated for 12 hours in continuous light with an intensity of 100 µmol photons/m^2^/s. Gas samples were withdrawn, and ethylene production was measured using a Gas Chromatograph (Agilent 6890 N) equipped with a Poropak N column (inner dimensions 5′×I/8″) and a flame ionization detector using argon as the carrier gas (flow rate of 65 ml/min), according to the manufacturer's instructions. The temperature of the injector, detector, and oven were 150, 200 and 100 °C, respectively.

### Determination of chlorophyll

Chlorophyll content was measured spectrophotometrically at 562 nm and 665 nm on an Olis DW2000 conversion (Online Instrument Systems) after extraction with 100% methanol. The amount of chlorophyll *a* per mL culture was calculated according to [19].

### Microarray analysis

The microarray was designed based on the complete *Cyanothece* 51142 genome sequence information [20]. Single 60-mer oligonucleotide probes were generated for each of the 5,304 ORFs and spotted as triplicates on the microarray, to provide an estimate of intraarray variance. Agilent Technologies produced the arrays. RNA preparation and microarray hybridization procedures were performed as previously described [13]. For each experiment, three replicate microarrays were analyzed per time point, with two biological replicates and one dye swap, for a total of 9 genome equivalent microarrays per time point.

### Identification and classification of differentially expressed genes

The microarray data analyses were conducted using the Matlab software package (MATLAB version 7.6, MathWorks Inc.). Numerical ratios of gene expression were obtained by computing the gene expression levels at 8% versus 0.03% CO_2_ for each gene at each time point. These ratios were adjusted using LOWESS algorithm to obtain normalized values. Differentially expressed genes showed expression levels that changed by more than 1.5 fold at a significance level of 1% in at least one of the time points. Early and late responsive genes were grouped based on their expression profiles over the entire time course. Early or transient responsive genes showed differential expression only during the first three time points, whereas late responsive genes revealed differential expression only during the remaining time points. To identify long term behaviors of genes that are either induced or repressed by high CO_2_ concentrations, late response genes were classified into 16 different groups using k-means clustering according to [21] (Figure S1). For this analysis, expression levels of genes at the time points L6 and D6 from both days were considered. Genes in groups (a)–(f) are significantly up-regulated under 8% CO_2_ while those in the remaining groups are significantly down-regulated.

In order to understand the overall effect of CO_2_ on diurnal rhythm, expression data were qualitatively compared to data from a previous study performed under alternating light/dark conditions [13]. For genes with overall higher expression levels under 8% CO_2_, the following four main behaviors could be identified: (1) Genes that are always up-regulated under high CO_2_. (2) Genes that are significantly induced under high CO_2_ (fold change >1.5) at the time of their active transcription. These genes show a fold change >1.5 at either L6 or D6 consistent with their active transcription either during the light or dark period. (3) Genes that are significantly up-regulated (fold change >1.5) under 8% CO_2_-enriched air at the same time points on both days. (4) Genes that are significantly induced (fold change >1.5) under 8% CO_2_ conditions in at least one time point without any significant change at other time points. Genes with reduced transcript levels under 8% CO_2_ were similarly classified.

### Semi-quantitative reverse transcription (RT–PCR)

Semiquantitative RT–PCR analyses were performed on RNA samples isolated from cultures grown under aeration with ambient and 8% CO2-enriched air 42 h after the step down (time point D6, Figure S2). RNA was isolated and quantified as previously described [13]. A total of 700 ng DNase (Promega)-treated RNA was used for reverse transcription with the Superscript II Reverse Transcriptase and random primers (Invitrogen) according to the manufacturer's instructions. The absence of DNA contamination was tested for each RNA sample. PCR was carried out at 94 °C for 2 min, followed by 94 °C for 30 s, 58 °C for 20 s, 72 °C for 20 s and a final extension time of 2 min at 72 °C. A total of 23 cycles for *nifH* using the following primer pairs F: 5′-ACCATTGCTGCGTTAGCTGAAAC-3 and R: 5′-TAATACCACGACCCGCACATCCA-3′; 22 cycles for *hupS*; F: 5′-ATAGCTGGTTTCGTTGTCGCTGT-3′, R: 5′-CGAAGTCTTGGGTGGTTGCTTTG-3′; *glgA1* F: 5′-ATATGTGGGTCGCCTTGATGACC-3′, R 5′-TTCACTAAACCTCCTACGCCACG-3′; *cpcA* F; GTGCTGCCAACGCAGTTTATCAG-3′, R: 5′-AGGAGTTGGCTTCTACAGCAGGA-3′; and 25 cycles *16 S RNA* F: 5′- AGAGGATGAGCAGCCACACT-3′, R: 5′-TAATTCCGGATAACGCTTGC-3′.

### O_2_ evolution and O_2_ uptake

The capacity of oxygen evolution and oxygen uptake from *Cyanothece* 51142 cells was determined using a Clark-type electrode during the middle of the light and dark period, respectively. The measurements correspond to the time points L6 (6 hours into the light period) and D6 (6 hours into the dark cycle) at 30 and 42 hours after the step down. Oxygen evolution measurements were conducted at a light intensity of 8,250 µmol photons/m^2^/s. The amount of oxygen released or taken up was calculated per Chl *a*.

### Determination of cell size and cell numbers

Cell size measurements on cultures grown under ambient CO_2_ and 1% and 8% CO_2_-enriched air were performed at time point L6 (6 h into the light cycle) for *Cyanothece* 51142 grown in 12 h light/dark using a Nikon Eclipse 80 i microscope equipped with 100× oil immersion objective (Figure S3). Cell length of more than 800 cells per treatment was measured from photomicrographs using the Metaview analysis program, and the cell size distributions were compared using the Two-sample Kolmogorov-Smirnov test at a significance level of 0.1%. Cell numbers were determined using a Nexcelom Bioscience Cellometer Auto M10 according to the manufacturer's instruction.

### Electron microscopy

*Cyanothece* 51142 cells were prepared for electron microscopy as previously described [22]. Cultures were grown as described above for 7 days, harvested by centrifugation and resuspended in a small volume, loaded into planchettes and frozen in a Baltec high pressure freezer (Bal-tec). Samples were freeze substituted in 2% osmium/acetone and embedded Epon/Araldite. Thin sections (∼80 nm) were stained with uranyl acetate and lead citrate, and imaged using a LEO 912AB electron microscope equipped with a ProScan digital camera.

### Data deposition

The microarray data have been deposited in GEO (http://www.ncbi.nlm.nih.gov/geo) under the following accession number: GSE37621.

## Results and Discussion

### Identification of differentially expressed genes under high CO_2_ conditions

**Figure 1:**
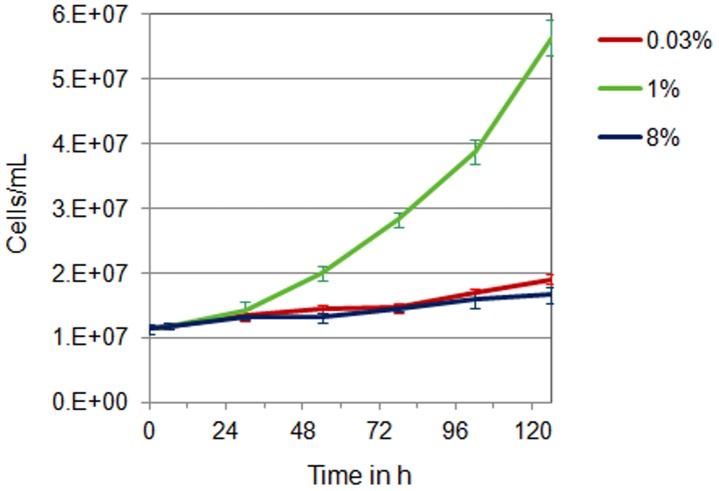
*Cyanothece* 51142 cultures grown under 0.03%, 1%, and 8% CO_2_-enriched air. Growth was determined based on the increase in cell number over time and measured at time point L6 (6 h into the light cycle) over a period of 6 days. Each data point represents the average count value from three biological replicates. Error bars indicate S.D. values from the average.

**Figure 2:**
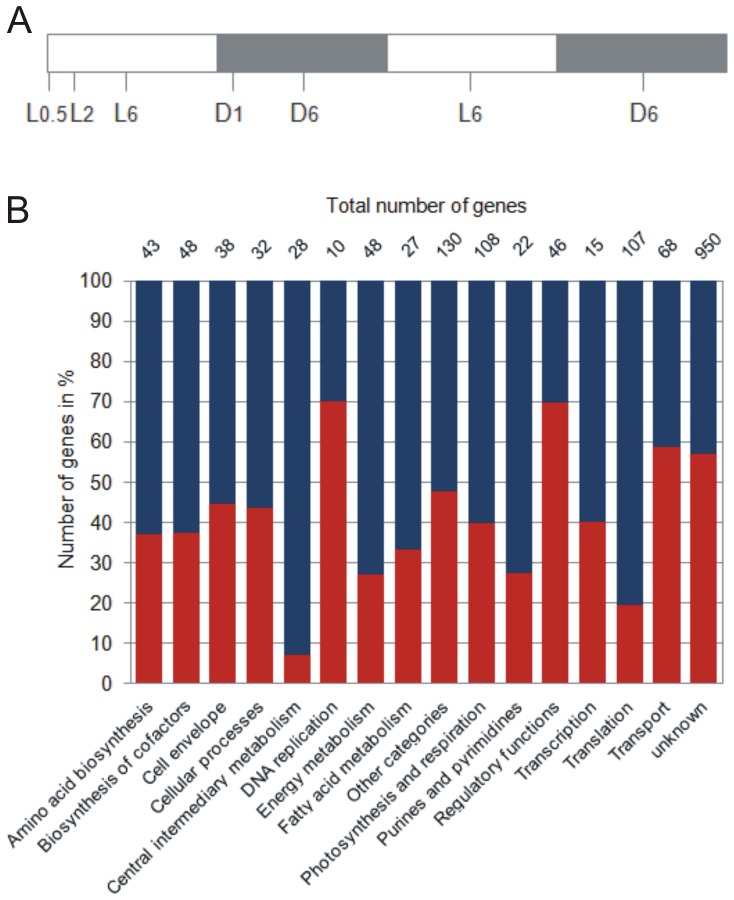
Experimental overview and functional category breakdown of differentially expressed genes with higher and lower transcript levels in 8% CO_2_ conditions. (A) Sample collection of nitrogen fixing *Cyanothece* 51142 cultures after step down from 8% CO_2_-enriched to ambient CO_2_ conditions. Time points were collected at L0.5, L2, L6, D1, D6, L6, and D6 for 30 min, 2 h, 6 h, 13 h, 18 h, 30 h and 42 h after the transition to ambient CO_2_ during the light (L) or dark (D) period. (B) The log_2_ ratios of individual genes were compared to the log_2_ ratios obtained from a previous study under alternating 12 hours light/dark [13] to correlate their response to CO_2_ with the phase of their active transcription. The percentage of up-regulated (blue) and down-regulated (red) genes under high CO_2_ was calculated based on the total number of differentially expressed genes in each functional category.

Initial growth experiments using ambient CO_2_ (0.03%), elevated CO_2_ (1%), and high CO_2_ (8%) revealed that 1% CO_2_ considerably enhances growth of *Cyanothece* 51142 cultures (Figure 1). This finding is consistent with previous observations in other diazotrophic cyanobacteria [9] and is presumably the result of increased CO_2_ assimilation rates. In contrast, nitrogen fixing cultures of *Cyanothece* 51142 aerated with ambient and 8% CO_2_ grow at similar but significantly lower rates as compared to 1% CO_2_ (Figure 1). This result suggests that high CO_2_ conditions generate a nutritional and/or energetic imbalance that outweighs the beneficial effect of elevated CO_2_ on the growth rate. In order to understand those CO_2_ mediated metabolic changes, we investigated the *Cyanothece* 51142 transcriptome for the effect of high CO_2_ using whole-genome DNA microarrays. An overview of the experimental design is given in Figure 2A. In total 2086 genes, that changed by at least 1.5-fold (p-value <0.01, Table S1) could be identified. 54 of them showed differential expression only at the first three time points, 1672 genes were found to be responsive exclusively at later time points, and 360 genes were differentially expressed during the entire time course. The majority of early responsive genes are either associated with transport functions, which include the phosphate binding protein *ptsS1* (*cce_1859*), as well as the ferric uptake regulator *fur2* (*cce_1951*), or belong to genes of unknown functions (Table S1). A further classification revealed 875 genes that are up-regulated and 851 genes that are down-regulated under high CO_2_ (Figure 2B, Figure S1, Table S2). The functional category breakdown showed that the majority of genes with higher transcript levels in 8% CO_2_-enriched air are associated with assigned functional categories (Figure 2B). Among them are especially genes related to the central intermediary metabolism, energy and fatty acid metabolism, purine and pyrimidine metabolism as well as ribosome biogenesis and translation. In contrast, the majority of genes involved in regulatory cell functions, DNA metabolism and transport show lower expression levels in 8% CO_2_. Notably, almost all genes associated with photosynthesis exhibit reduced transcript levels whereas genes related to respiration are up-regulated in 8% CO_2_ conditions (Table S2). This strict separation of distinct cellular processes indicates modified metabolic requirements under conditions of high CO_2_ that affect both phases of the diurnal cycle.

### CO_2_ alters the ultrastructure of the cells

**Figure 3:**
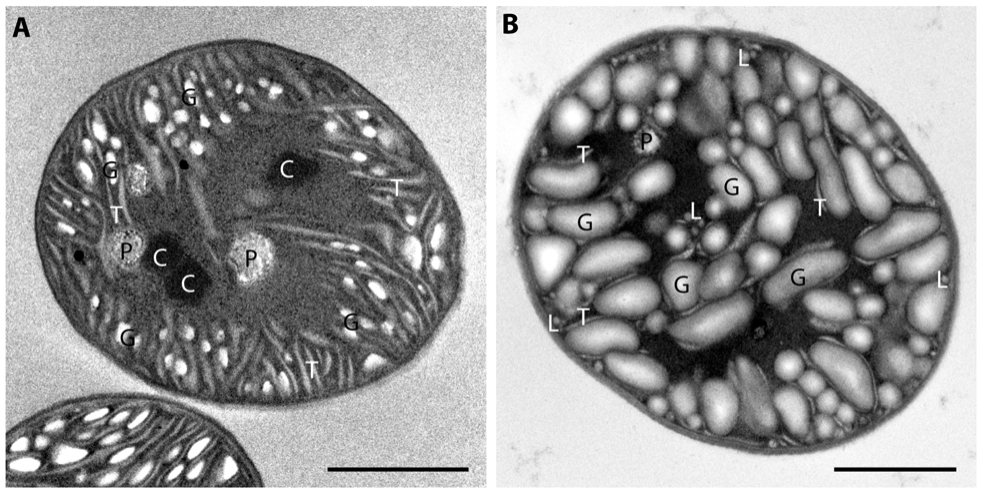
Electron micrographs of Cyanothece 51142. The cells were grown in ambient (A) and 8% CO_2_ (B) under 12 h light/dark conditions and harvested at L10. Labeled are carboxysomes (C), polyphosphate bodies (P), glycogen granules (G), lipid bodies (L), and thylakoid membranes (T). Bar = 1 µm.

A detailed examination of cells grown in high CO_2_ by transmission electron microscopy showed a dramatic change in size and number of glycogen granules (Figure 3). In cells grown under ambient conditions, glycogen granules are ∼150 nm in length and accumulate between thylakoid membranes [22]; however, under high CO_2_ conditions, glycogen granules can be larger than 500 nm, and fill much of the central cytoplasmic region of the cells. This accumulation of glycogen granules results in a crowded environment for thylakoid membranes and other cellular components such as carboxysomes. In our examination of over 50 cells grown under high CO_2_ conditions, we were not able to unequivocally identify any carboxysomes in the cells, suggesting that carboxysomes are rarely formed under these conditions.

### Elevated CO_2_ results in production of carbon-rich cellular components

**Figure 4:**
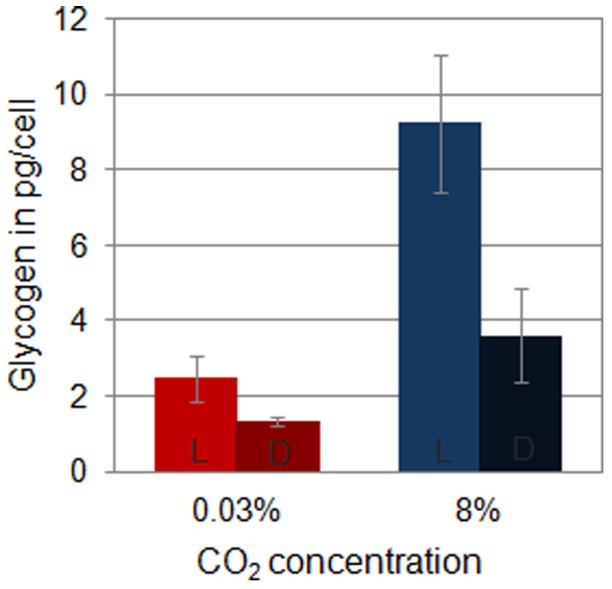
Glycogen content of *Cyanothece* 51142 cells grown under ambient and 8% CO_2_. The glycogen content per cell was determined at time point L6 (6 h into the light cycle) and time point D6 (6 h into the dark cycle). Each column represents the average value from three biological replicates. Error bars indicate S.D. values from the average.

**Figure 5:**
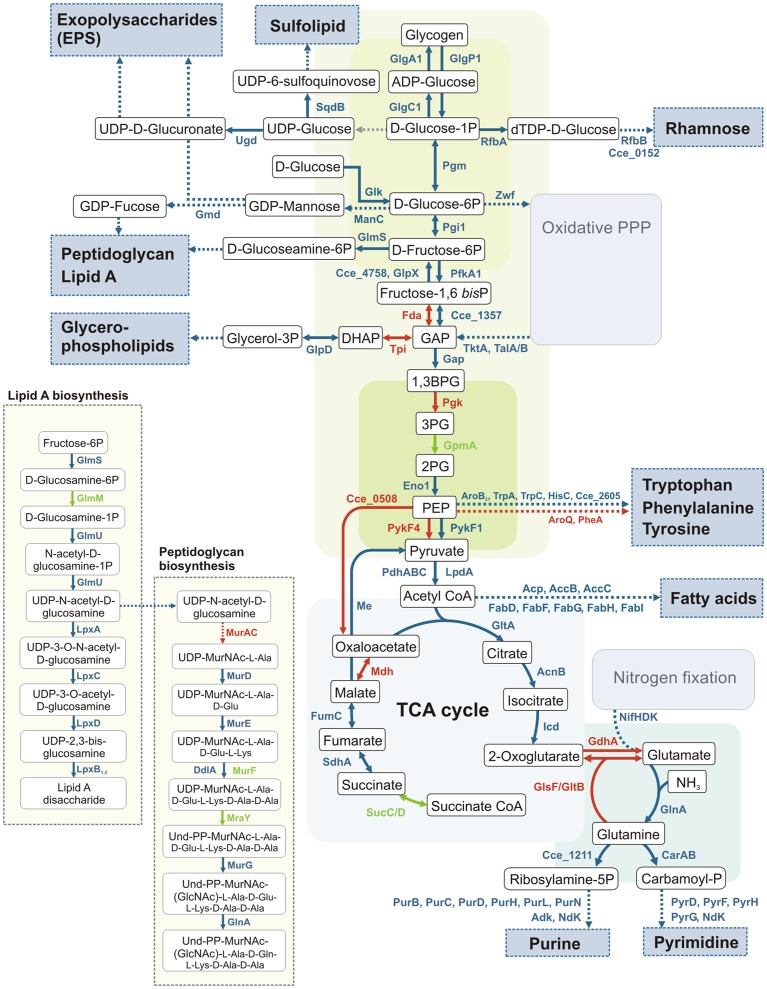
Schematic overview of carbon metabolism related processes in *Cyanothece* 51142. Enzymatic steps involving genes with maximal transcript abundances under 8% CO_2_ are shown in *blue* while down-regulated genes are represented by *red* arrows. *Green* colored arrows show genes that are not differentially expressed. Dashed lines represent more than one associated enzymatic reaction. The corresponding gene expression data are summarized in Table S2.

Genes involved in the synthesis of glycogen are up-regulated under 8% CO_2_-enriched air, which is consistent with an increase in the glycogen content per cell in *Cyanothece* 51142 cultures grown with 8% CO_2_-enriched air (Table S2, Figures 3 and 4). The accumulation of glycogen, as well as the production of extracellular polysaccharides (EPS) under carbon-rich conditions, is well documented [23], [24]. Different studies on the structure of cyanobacterial EPS indicate the presence of acidic hexoses, neutral hexoses, pentoses and desoxyhexoses in varying combination and ratios [23]. Many genes involved in the synthesis of potential EPS precursors, such as glucose, mannose, fucose, fructose, rhamnose and glucuronic acid are up-regulated at the transcript level under high CO_2_ conditions (Table S2, Figure 5). Although both polymers gather large quantities of the cellular available carbon, their metabolic fate differs significantly because the synthesis of glycogen contributes to the overall storage whereas the production of EPS results in the overall loss of cellular carbon. A potential role for EPS in releasing excess cellular carbon would, in combination with a previously suggested role in protecting the nitrogenase enzyme from exogenous oxygen [25], provide a mechanism to adjust the cellular C/N ratio in favor of nitrogen.

In addition, the sugar monosaccharides fucose and mannose are required for the production of peptidoglycan and lipopolysaccharides [26]. Both polymers are critical structural components of the cyanobacterial cell wall and are necessary for bacterial growth [27]. Notable is the coordinated up-regulation of genes involved in these pathways (Figure 5), which suggests an elevated cell wall synthesis.

*Cyanothece* 51142 belongs to the group of rod shaped bacteria that after cell division enter into a phase of peptidoglycan synthesis that supports lateral cell wall expansion. The bacterial actin homolog MreB (*cce_2000*), which plays a central role in this process, reveals higher transcript levels under high CO_2_ conditions and thus fosters the idea of an enhanced lateral cell growth. In addition, the transcripts of the bacterial tubulin FtsZ (*cce_1314*), which orchestrates peptidoglycan synthesis at the mid-cell to initiate cell division, are also more highly abundant under 8% CO_2_-enriched conditions. Both MreB and FtsZ require several proteins to facilitate the process of peptidoglycan synthesis. Among them are the rod shape-determining protein RodA (*cce_0922*), the transpeptidases PBP2 (*cce_3458*) and PBP3 (*cce_3444*), the glycosyltransferases PBP1A (*cce_2453*) and PBP1B (*cce_4147*), with PBP1A to be appointed to the sidewall and PBP1B to the divisome [28]. With the exception of RodA, all proteins show higher abundances at the transcriptional level under high CO_2_ conditions (Table S2).

### High CO_2_ leads to an increase in cell size

**Figure 6:**
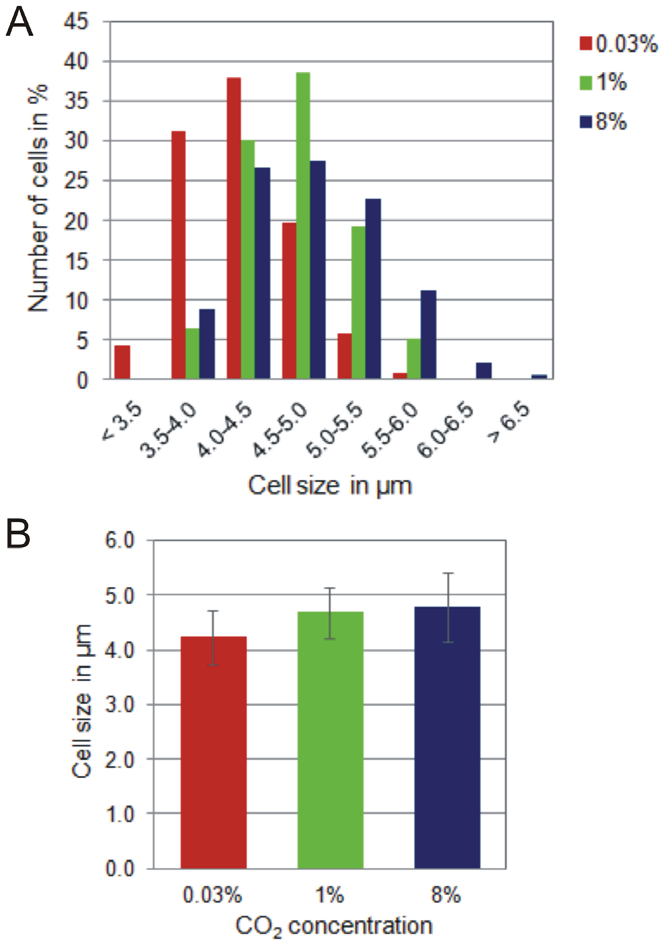
Cell size and cell size distribution of *Cyanothece* 51142. *Cyanothece* 51142 cells grown under 0.03%, 1% and 8% CO_2_-enriched air were assayed for changes in (A) cell size and (B) cell size distribution. Each column in (A) represents the average value from measurements of more than 800 cells from three biological replicates. Error bars indicate S.D. values from the average. The cell size distribution of more than 800 cells for each growth condition in (B) is shown in percent and based on the total number of cells within each cell size range.

Growth experiments of *Cyanothece* 51142 revealed a significant increase in cell size under 8% CO_2_-enriched air (Figure 6, Figure S3). This is in agreement with the impact of high CO_2_ on transcript levels of genes involved in peptidoglycan and lipopolysaccharides biosynthesis as well as translation (Table S2). A total of 86 genes, mostly encoding for ribosomal proteins, are up-regulated at the transcriptional level. This concerted response is significant because ribosomal protein translation is one of the key events for cell growth and biomass production. Earlier studies in *Synechocystis* 6803 and higher plants pointed to a correlation between transcript abundance of ribosomal genes and cell growth [11], [29], [30]. In addition, different genes known to be engaged in translation such as *gatABC* (*cce_1710*; *cce_1674*; *cce_0284*), the translation initiation factor *infA* (*cce_4035*) and the elongation factors *efp* (*cce_2122*), *tsf* (*cce_0704*), *fus1* (*cce_4089*), *fus2* (*cce_2051*) and *tufA* (*cce_4088*) are also more highly abundant under high CO_2_ conditions.

This study suggests that high CO_2_ conditions control not only metabolic but also cell cycle processes. Alterations in cyanobacterial biomass production and growth that occur in response to environmental changes have typically been reported as changes in cell number and/or fresh and dry weight. Other aspects of the cell cycle process such as cell size have been largely disregarded. However, the involvement of CO_2_ as a potential key player in regulating cell size and morphology became evident in previous studies of *Trichodesmium* IMS101, which revealed an increase in trichome length under elevated CO_2_ conditions [9]. Similar results have been obtained using fructose as exogenous carbon sources in *Anabaena variabilis* [31] and suggest a direct link between carbon metabolism and cell growth. Despite the pivotal role of carbon as a macronutrient and an essential building block for virtually all cellular components, an increasing number of studies put forward a potential function of CO_2_ and CO_2_-derived carbon metabolites as signaling molecules that control fundamental processes in cell growth. In fact, *Cyanothece* 51142 growth under 1% CO_2_-enriched air considerably enhances cell size while cells divide at faster rates (Figures 1 and 6) and thus likely excludes a possible stress response which has previously been related to the occurrence of larger cells in cyanobacterial populations [32]. This observation highlights the crucial role of CO_2_ for cell growth and proliferation by altering both cell size and doubling time. The ability to adjust cell size under carbon-rich conditions suggests a link between cell growth and cellular carbon levels beyond the evolutionary concept to reproduce at faster rates in nutrient-rich environments.

The metabolic branch points and regulatory factors that coordinate carbon metabolism with cell size control still remain unknown in cyanobacteria. However, the fact that the majority of genes related to cell wall synthesis and cell division are up-regulated under high CO_2_ conditions suggests either an increase in the activity of cell wall synthesizing enzymes and/or the presence of molecular factors that regulate cell division depending upon carbon availability.

### CO_2_ regulates the activity of different central metabolic processes

**Figure 7:**
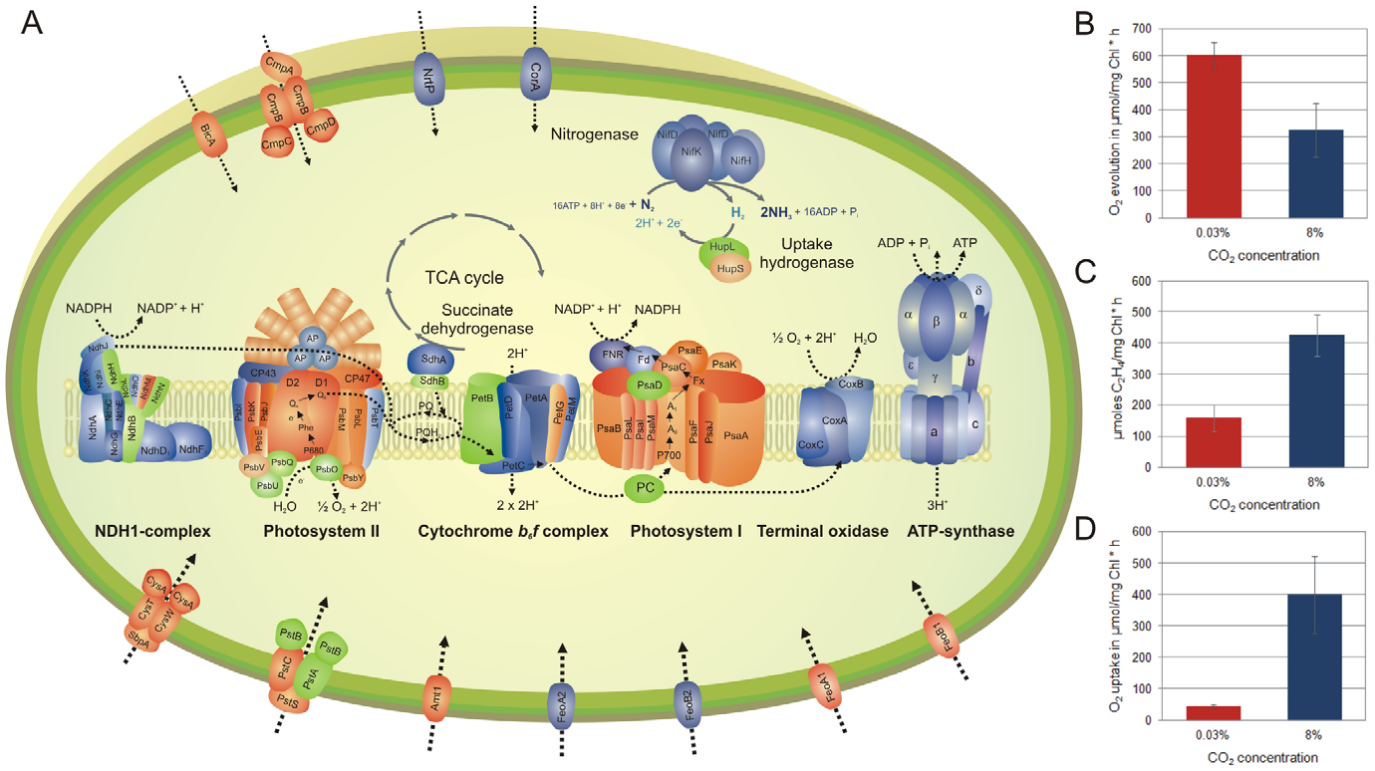
Overview of differentially expressed genes encoding for structural subunits involved in photosynthesis, respiration, nitrogen fixation and transport, and measurements of corresponding metabolic activities. (A) Protein subunits encoded by genes with higher expression levels under 8% CO_2_-enriched conditions are shown in *blue* color. *Red* color indicates proteins encoded by genes that are down-regulated under high CO_2_. *Green* colored protein subunits represent genes that are not differentially expressed. Respiratory and photosynthetic electron transport chains are illustrated according to [42], and the composition of the NDH1 complex according to [43]. The corresponding gene expression data are summarized in Table S2. (B) The capacity of oxygen evolution at time point L6, (C) nitrogenase activity assayed as acetylene reduction at night and (D) respiration at time point D6. The graphs contain averaged values obtained from three biological replicates. Error bars represent the S.D. from the average.

The apparent increase in the C/N ratio under conditions of high CO_2_ necessitates cellular adjustments to cope with the modified nutritional requirements while maintaining the overall metabolic rhythms during the diurnal cycle. Thus, high levels of CO_2_ reduce the photosynthetic activity and lead to a marked down-regulation of transcripts related to photosynthesis once the carbon reservoir is replenished (Figures 7A and B, Table S2). Such feedback regulations of photosynthesis through products of primary metabolism have also been described for higher plants [33] and could either be the result of an overall reduction in photosynthetic activity or the consequence of a shortened phase of activity.

However, notable is not only the concerted down-regulation of genes involved in photosynthesis, but also the lower levels of transcripts associated with CO_2_ and bicarbonate uptake (Figure 7A, Table S2).

In addition, genes encoding for proteins involved in phycobilisome degradation, like NblA1/2 (*cce_0099*, *cce_0100*) and the ATP-dependent Clp proteases ClpC1 (*cce_4247*) and ClpP3/4 (*cce_1910*, *cce_1911*) [34] are up-regulated under 8% CO_2_ (Table S2). The degradation of phycobiliproteins couples the regulation of photosynthetic activity to the acquisition of nutrients by reducing the photosynthetic antenna size and, as a result, increasing the cellular nitrogen pool while diminishing further carbon input. Thus, under diazotrophic conditions, readily available CO_2_ mandates the reallocation of nitrogen from other cellular sources to adjust the high cellular C/N ratio during the light period. Although the degradation of phycobilisomes is a well-studied phenomenon in cyanobacteria under nitrogen limitation, exploiting this capacity to balance changes in nutritional requirements by adjusting metabolic activities provides an elegant strategy to regulate diurnal fluctuations in the cellular carbon and nitrogen pools.

**Figure 8:**
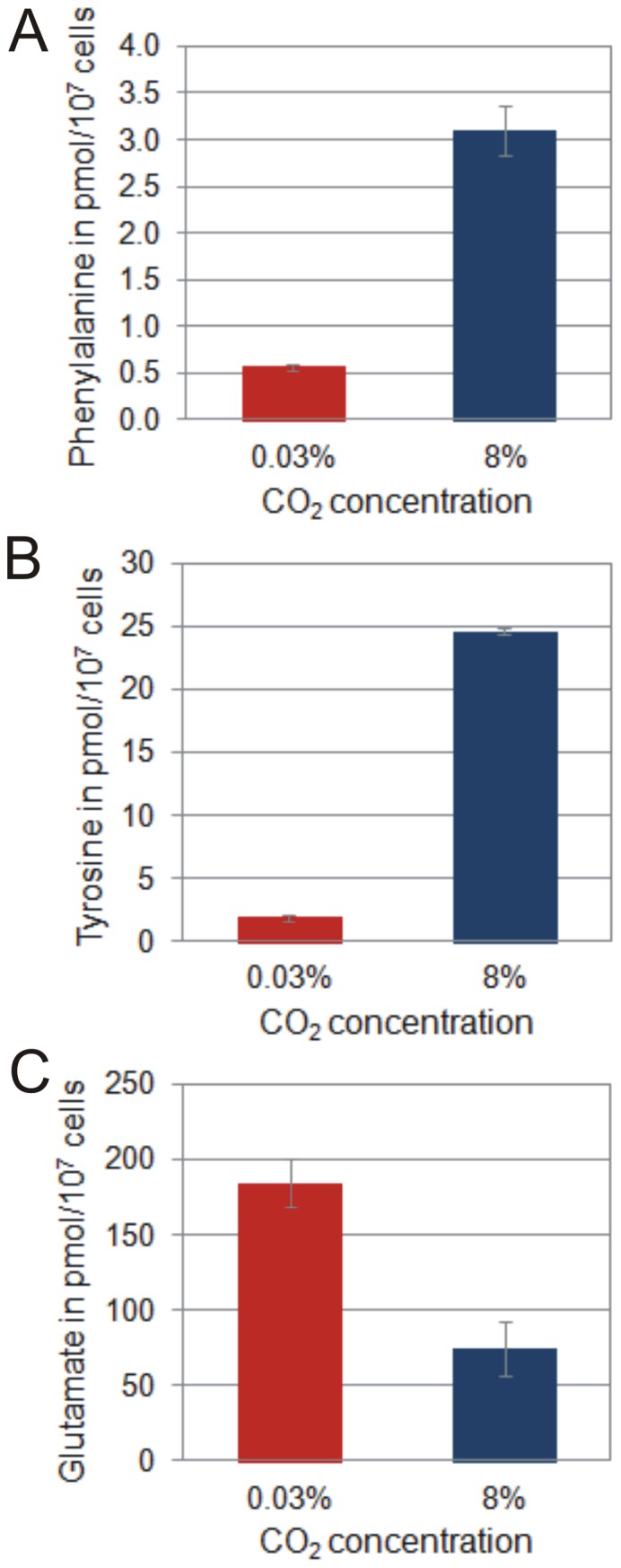
Cellular amino acid concentrations of diazotrophically grown *Cyanothece* 51142 cells under ambient and high CO_2_ conditions. The cellular phenylalanine, tyrosine and glutamate concentrations were measured at time point D6. Error bars represent S.D. of two separate measurements from two biological replicates.

In contrast, genes involved in nitrogen fixation are significantly up-regulated (Figure 7A, Table S2) and result in higher nitrogenase activities under CO_2_-rich conditions at night (Figure 4C). Higher rates of nitrogen fixation illustrate not only an increased demand for nitrogen but also an intracellular environment suitable for high nitrogenase activities. Potential sinks for newly fixed N_2_ are different amino acids that serve as precursors for a variety of nitrogen containing metabolites. The pyruvate kinase PykF1 (*cce_3420*), one of the key enzymes in regulating carbon flow towards amino acid biosynthesis, shows higher expression levels in 8% CO_2_-enriched air. Higher intracellular concentrations of phenylalanine and tyrosine, as well as higher expression levels of genes involved in their synthesis, have been observed in *Cyanothece* 51142 grown with 8% CO_2_-enriched air (Figure 8A and B). In contrast, although contributing highest to the cellular amino acid pool, the amount of glutamate under ambient conditions significantly exceeds the level found in cells grown with high CO_2_ (Figure 8C). Glutamate has previously been shown to inhibit diazotrophic growth, heterocyst differentiation and nitrogenase activity in *Nostoc* ANTH [35]. In addition, studies in *Phormidium laminosum* showed that under conditions of nitrogen starvation glutamate levels remain high and are utilized for amino acid synthesis when nitrogen becomes available [36]. Similar results have been observed in *Gloeothece* under nitrogen fixing conditions [37].

Glutamate is not only an essential precursor for the synthesis of other amino acids, but also for tetrapyrroles and nucleic acids. Different glutamate utilizing enzymes such as the glutamyl-tRNA synthetase GltX (*cce_1758*) as well as HemA (*cce_3976*) and HemL (*cce_0172*), all of which are involved in the synthesis of the chlorophyll precursor 5-aminolevulinic acid, reveal higher transcript abundances under elevated CO_2_ conditions during the dark. The potential to synthesize chlorophyll in the dark, owing to the existence of a light-independent isoform of an otherwise light-dependent protochlorophyllide reductase, distinguishes cyanobacteria from the majority of higher plants. However, although the synthesis of 5-aminolevulinic acid and chlorophyll in anticipation of the subsequent photoperiod seems intuitive, it remains to be determined whether the increase in cellular glutamate levels under high CO_2_ conditions stimulates these processes.

In addition, a significant number of genes involved in the synthesis of nitrogen containing bases are up-regulated under 8% CO_2_ (Table S2; Figure 5). The demand for *de-novo* synthesis of purines and pyrimidines increases particularly in growing and dividing cells, where processes such as DNA replication, RNA synthesis, and the production of ribosomal and transfer RNA for protein biosynthesis govern cell metabolism. Purine biosynthesis is known to play a crucial role in the primary nitrogen metabolism of different legumes by assimilation and detoxification of newly synthesized NH_3_ [38]. Furthermore, as essential precursors for coenzymes such as NAD, NADP, FAD, SAM and coenzyme A as well as for co-substrates in the form of UDP- and ADP-glucose, nucleotides also participate in the synthesis of polysaccharides, glycoproteins and phospholipids. The concerted up-regulation of genes involved in the *de-novo* synthesis of different amino acids, nucleotides and pigments in synchrony with genes involved in nitrogen fixation during the dark suggests that these processes are primary sinks for newly fixed nitrogen in *Cyanothece* 51142 and as such are especially important under conditions of high CO_2_.

In addition, the cyanophycin synthetase *cphA* (*cce_2237*) reveals elevated transcript levels in CO_2_-rich conditions. Cyanophycin is a nitrogen-rich non-ribosomally synthesized peptide composed of multi-L-arginyl-poly-L-aspartate that plays a crucial role in the transient storage of fixed nitrogen. It serves as dynamic reservoir under conditions of nitrogen limitation [39], [40].

Thus, changes in the transcript abundance of genes involved in the biosynthesis and degradation of different metabolites are coordinated to accommodate daily fluctuations in the availability of essential nutrients. In line with this notion are the high transcript levels of heme oxygenases *ho1* (*cce_2573*) and *ho2* (*cce_2324*) under elevated CO_2_ at night. Those enzymes initiate the synthesis of phycobilin once nitrogen in the form of NH_3_ becomes metabolically available.

Our data also show that elevated CO_2_ guides the concerted up-regulation of transcripts related to respiration. The majority of genes involved in respiration are up-regulated under CO_2_-rich conditions, which results in higher respiration rates (Figures 7A and D). The boost in respiration during the dark period ensures a continuous flow of carbon, energy and electrons for downstream processes such as nitrogen fixation while depleting the cellular carbohydrate storage to enable the accumulation of assimilates during the next photoperiod (Figure 4). Our analyses suggest that the complex regulatory interactions of photosynthesis, respiration and nitrogen fixation converge at the level of carbon metabolism. In fact, a possible role for storage carbohydrates in regulating the respiratory activity has been discussed for soybeans grown under elevated CO_2_ [41]. Carbohydrate accumulation and degradation reflect not only the efficiencies of photosynthesis and respiration, but also the capacity to utilize the breakdown products in downstream metabolic processes. Figure 5 summarizes the main carbon-based metabolic processes and highlights the fact that the majority of associated genes reveal higher transcript levels in 8% CO_2_. However, whether additional available carbon sources and energy are channeled towards growth also depends on the availability of other essential nutrients such as nitrogen. The high demand for nitrogen under high CO_2_ conditions is likely to affect cell growth because a significant amount of the metabolically derived energy has to be devoted for nitrogen fixation.

## Summary

This analysis establishes the view that an excessive supply of CO_2_ and high C/N ratios limits the overall capacity for carbon uptake while stimulating carbon releasing and/or consuming activities. We found that adaptation to and growth under high CO_2_ is intertwined with modifications of temporal metabolic activities that govern diurnal rhythms in *Cyanothece* 51142. This analysis shows further that daily rhythms of carbohydrate accumulation and degradation reflect not only the efficiencies of photosynthesis and respiration under high CO_2_ conditions, but also the cellular ability to accommodate unbalanced C/N ratios with metabolic sink activities.

## Supporting Information

Figure S1 **Gene clusters of differentially expressed genes.** Based on their expression profiles at the time points L6 and D6 from both days, the genes were clustered into 16 different groups. The groups (a)–(f) contain all genes that are significantly up-regulated under 8% CO_2_ and the groups (g)–(p) include all genes that are significantly down-regulated under 8% CO2.
(TIF)

Figure S2 **Reverse transcription PCR analysis of differentially expressed genes.** The transcript abundances of different genes involved in nitrogen fixation (*nifH*, *hupS*), carbon metabolism (*glgA1*) and photosynthesis (*cpcA*) were measured at time point D6 (6 hours into the dark cycle) in samples isolated from cells grown under ambient and high CO_2_. 16 S rRNA was used as the loading control.
(TIF)

Figure S3 **Light micrographs of** ***Cyanothece*** **51142.** The cells were grown under ambient (A) and 8% CO_2_ (B) under 12 h light/dark conditions. Bar = 5 µm.
(TIF)

Table S1 **Dataset of early and late responsive genes.** The table contains all genes which changed by at least 1.5-fold over the entire time course. The time points are labeled as L0.5, L2, L6, D1, D6, L6, and D6 for 30 min, 2 h, 6 h, 13 h, 18 h, 30 h and 42 h after the transition to ambient CO_2_ collected either during the light (L) or dark period (D). The genes were grouped into early and late responsive genes as well as genes that are differentially expressed during the entire time course. The values are given as average log_2_ ratios of transcripts from cells grown in 8% CO_2_-enriched air versus ambient CO_2_ for each time point.
(XLS)

Table S2 **Data set of differentially expressed genes under ambient and high CO_2_ conditions.** The table contains all differentially expressed genes that changed by at least 1.5 fold over the entire time course, and that are significantly up-regulated or down-regulated in 8% CO_2_-enriched air. The time points for samples collected either during the light (L) or dark period (D) are labeled as L6, D6, L6, and D6 for 6 h, 18 h, 30 h and 42 h after the transition to ambient CO_2_. Separate columns indicate the gene clusters of co-expressed genes, the overall behavior in 8% CO_2_ and alternating 12 h light/dark conditions. The values are given as average log_2_ ratios of transcripts from cells grown in 8% CO_2_-enriched air versus ambient CO_2_ for each time point.
(XLS)
