# Supplementary figures and images for: Carbon Availability Affects Diurnally Controlled Processes and Cell Morphology of Cyanothece 51142
Source: PLoS One. 2013 Feb 15;8(2):e56887. doi: 10.1371/journal.pone.0056887 (PMC3574086; doi:10.1371/journal.pone.0056887)

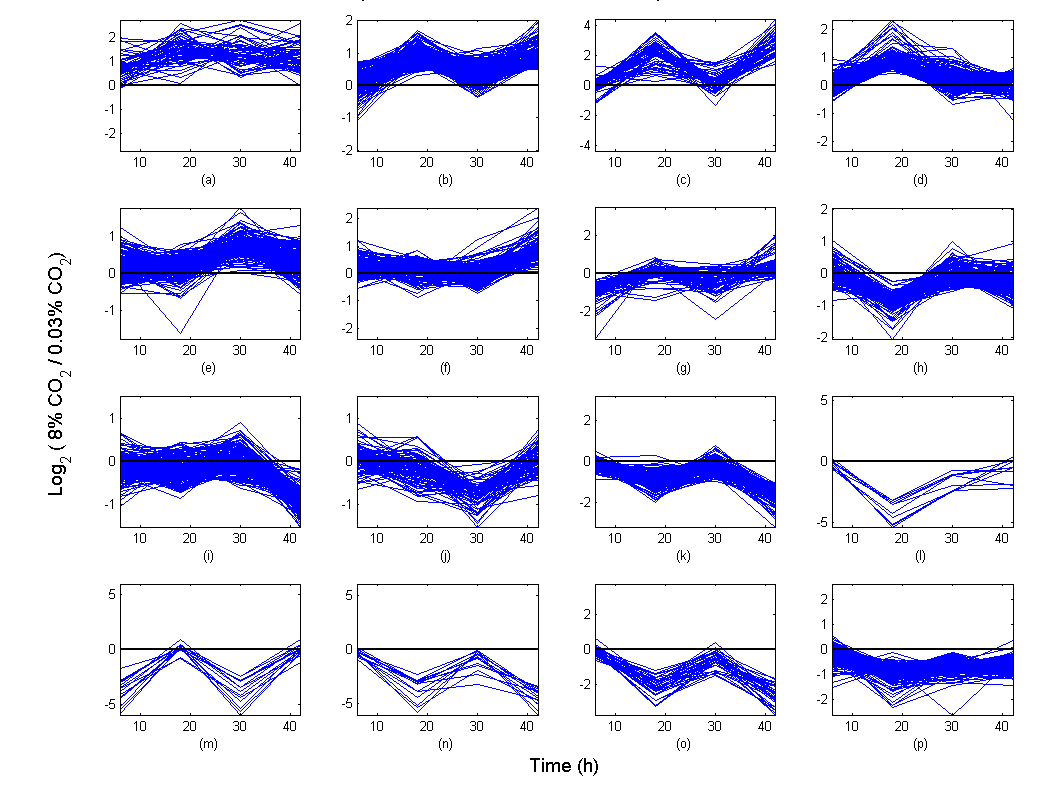

Supplement: Figure S1 — Gene clusters of differentially expressed genes. Based on their expression profiles at the time points L6 and D6 from both days, the genes were clustered into 16 different groups. The groups (a)–(f) contain all genes that are significantly up-regulated under 8% CO2 and the groups (g)–(p) include all genes that are significantly down-regulated under 8% CO2. (TIF) [file pone.0056887.s001.tif]

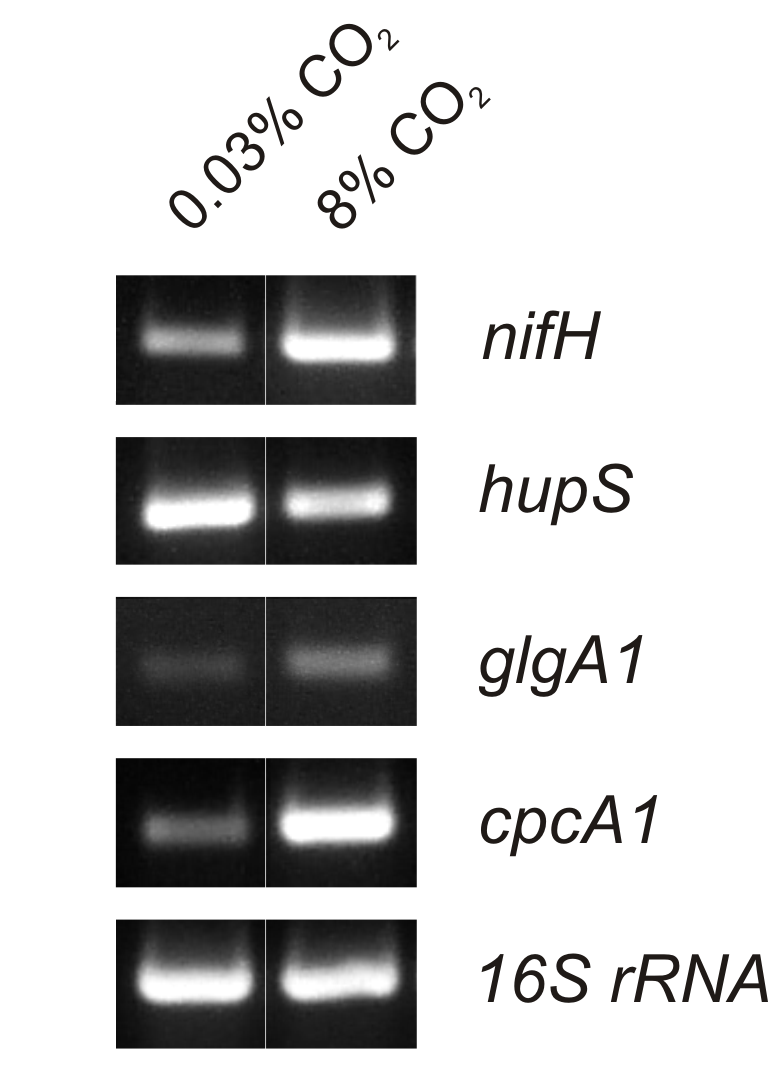

Supplement: Figure S2 — Reverse transcription PCR analysis of differentially expressed genes. The transcript abundances of different genes involved in nitrogen fixation (nifH, hupS), carbon metabolism (glgA1) and photosynthesis (cpcA) were measured at time point D6 (6 hours into the dark cycle) in samples isolated from cells grown under ambient and high CO2. 16 S rRNA was used as the loading control. (TIF) [file pone.0056887.s002.tif]

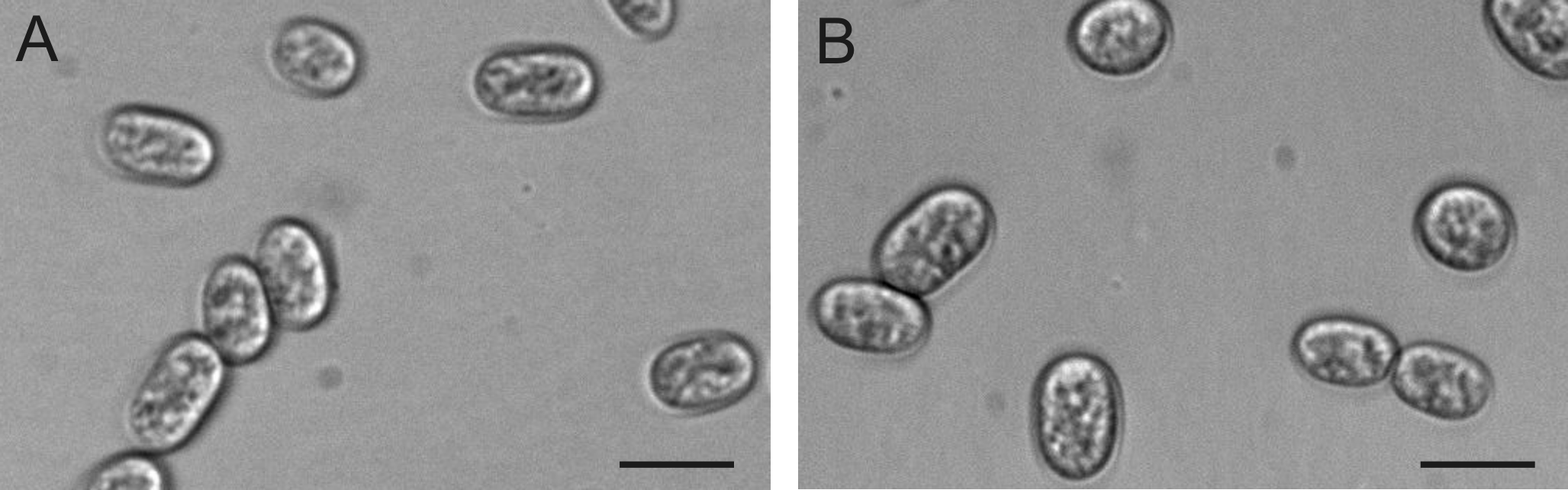

Supplement: Figure S3 — Light micrographs of Cyanothece 51142. The cells were grown under ambient (A) and 8% CO2 (B) under 12 h light/dark conditions. Bar = 5 µm. (TIF) [file pone.0056887.s003.tif]
